# Supplementary figures and images for: Ruxolitinib with resminostat exert synergistic antitumor effects in Cutaneous T-cell Lymphoma
Source: PLoS One. 2021 Mar 11;16(3):e0248298. doi: 10.1371/journal.pone.0248298 (PMC7951910; doi:10.1371/journal.pone.0248298)

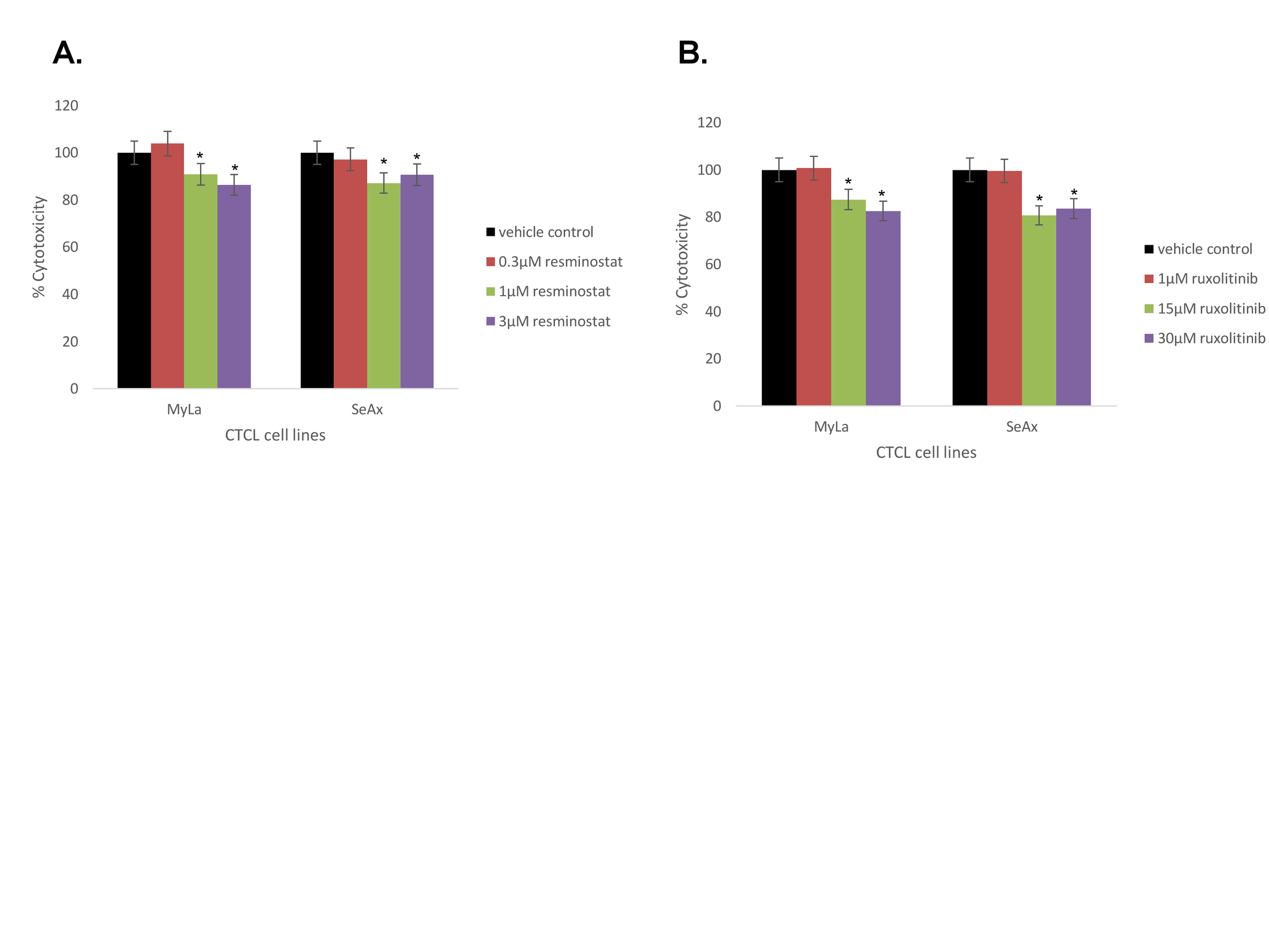

Supplement: S1 Fig — Values are the mean ± standard deviation of three experiments. *p <0.05, statistically significant differences versus untreated and single drugs. (TIF) [file pone.0248298.s001.tif]
